# Dual-Crosslinked Gelatin/Chondroitin Sulfate Hydrogel Loaded with Curcumin Liposomes for Multifunctional Wound Dressing

**DOI:** 10.3390/molecules31213868

**Published:** 2026-10-27

**Authors:** Jiatong Han, Pengfei Qiao, Daijie Wang, Hao Li, Liqiang Jin, Qinghua Xu

**Affiliations:** 1 Faculty of Light Industry, Qilu University of Technology (Shandong Academy of Sciences), Jinan 250353, China; 13165257918@163.com (J.H.); q1191493231@163.com (P.Q.); lihao@qlu.edu.cn (H.L.); 2 Food Resources Development and Health Product Creation International Joint Laboratory, Innovation Center for High-Value Development and Utilization of Bio-Health Food Technology of Shandong Province, Heze Branch of Qilu University of Technology (Shandong Academy of Sciences), Heze 274000, China; wangdaijie@qlu.edu.cn; 3 State Key Laboratory of Green Papermaking and Resource Recycling, Qilu University of Technology (Shandong Academy of Sciences), Jinan 250353, China

**Keywords:** gelatin, chondroitin sulfate, dual-crosslinked hydrogel, curcumin liposomes, wound dressing

## Abstract

Despite the widespread use of gelatin (Gel) in wound dressings due to its biocompatibility, biodegradability, and intrinsic bioactivity, it remains challenging to simultaneously achieve robust mechanical properties and multiple biofunctions in a single hydrogel system. Herein, we constructed a Gel/oxidized chondroitin sulfate (OCS) dynamic Schiff-base network and further introduced triglycidylamine (TGA) as a covalent crosslinker, yielding a Gel/OCS/TGA hydrogel with a proposed dynamic/covalent dual-crosslinked structure. Curcumin liposomes (Cur@Lipo), prepared using microfluidic technology, were then incorporated as bioactive nanofillers to obtain the multifunctional Gel/OCS/TGA/Cur@Lipo composite hydrogel. The hydrogel exhibited self-healing and injectable behavior, which may be associated with the reversible dynamic bonds present in the network. By adjusting the TGA content, the composite hydrogel achieved a tensile strength of 315 kPa, an elongation at break of 258%, and a compressive stress of 72 kPa at 60% strain. Moreover, the hydrogel exhibited sustained curcumin release, marked antioxidant activity (DPPH and ABTS radical scavenging rates of 59.8% and 83.98%, respectively), and antibacterial activity against *E. coli* and *S. aureus* (>99.99%), while maintaining good cytocompatibility. In summary, Gel/OCS/TGA/Cur@Lipo multifunctional composite hydrogel is a promising candidate for wound dressing applications, although further in vivo studies are required to confirm its clinical applicability.

## 1. Introduction

Upon skin injury, a series of complex wound healing processes are triggered, encompassing four overlapping and highly coordinated phases: hemostasis, inflammation, proliferation, and remodeling [1]. Adverse factors such as bacterial infection and excessive oxidative stress can severely disrupt this orderly process [2,3]. Therefore, it is imperative to select appropriate wound dressings to promote healing. Traditional wound dressings, such as gauze and sponges, can only provide physical coverage and exudate absorption, and are incapable of actively participating in or regulating the wound healing process [4]. Hydrogels, as polymeric materials with a 3D porous architecture, have attracted significant interest in the field of wound dressing in recent years due to their highly similar structure to the human extracellular matrix (ECM) [5].

Among various hydrogel matrix materials, gelatin (Gel), a natural polymeric material derived from the partial hydrolysis of animal collagen, exhibits unique advantages in the field of wound dressing owing to its excellent biocompatibility, biodegradability, and intrinsic bioactivity [6]. It retains abundant cell-recognition motifs from collagen, such as the arginine–glycine–aspartic acid (RGD) sequence, which facilitates cell adhesion, proliferation, and migration [7]. However, pure gelatin hydrogels are intrinsically limited by their poor mechanical properties, and a lack of active regulatory functions (e.g., antioxidant and antibacterial activities) over the wound microenvironment, thereby failing to meet the multifunctional demands of complex wound repair [8]. Moreover, hydrogel dressings are susceptible to damage from external mechanical stress during application, necessitating the incorporation of self-healing capability [9]. This property is commonly achieved through reversible dynamic covalent bonds within the hydrogel network, including imine bonds [10], boronate ester bonds [11], acylhydrazone bonds [12], Diels–Alder reactions [13], and disulfide bonds [14], among which imine bonds are the most widely applied due to their mild formation conditions and high reversibility [15]. Researchers have commonly combined gelatin with oxidized polysaccharides to construct dynamic crosslinked networks through Schiff-base reactions [16]. Among various natural polysaccharides, chondroitin sulfate (CS), a key component of the ECM, exhibits excellent anti-inflammatory activity by suppressing the overexpression of pro-inflammatory cytokines such as tumor necrosis factor-α (TNF-α) and interleukin-1β (IL-1β), as well as matrix metalloproteinases (MMPs), thereby alleviating the inflammatory response at the wound site [17]. Upon oxidation, the vicinal diol groups of CS are converted into aldehyde moieties (–CHO), yielding oxidized chondroitin sulfate (OCS) [18], whose aldehyde groups readily react with amino groups on gelatin to form dynamic Schiff-base bonds, conferring self-healing properties to the hydrogel [19].

Notably, a single dynamic crosslinking network provides only limited improvement in mechanical strength and fails to ensure long-term protection for wounds [20]. Incorporating chemical crosslinkers to construct a dual-crosslinked network is an effective strategy for enhancing the mechanical properties of hydrogels [21]. Triglycidylamine (TGA), a multifunctional, highly reactive, and bio-based small-molecule crosslinker, can react with the amino (–NH_2_), hydroxyl (–OH), and carboxyl (–COOH) groups on gelatin chains through its epoxy groups, forming a stable covalent crosslinked network [22]. Compared with epoxy compounds such as ethylene glycol diglycidyl ether, TGA exhibits good water solubility and higher reactivity [23]. Connolly et al. [24] applied TGA for the crosslinking modification of porcine aortic valve cusps and bovine pericardium used for bioprosthetic heart valves, achieving marked improvements in mechanical properties and biocompatibility. However, the use of TGA as a crosslinker for gelatin-based hydrogels remains unexplored. Inspired by its successful applications in biological tissue engineering, introducing TGA into the hydrogel network is expected to avoid the potential cytotoxicity associated with traditional crosslinkers (e.g., glutaraldehyde and EDC/NHS), while simultaneously enhancing the mechanical properties and structural stability without compromising its biocompatibility.

To achieve active modulation of the wound microenvironment, it is essential to endow the dressing with the capacity to scavenge excessive reactive oxygen species (ROS) and effectively inhibit bacterial infection [25]. Curcumin (Cur), a natural polyphenolic compound extracted from turmeric rhizomes, exhibits diverse biological properties, including antioxidant, broad-spectrum antibacterial, and anti-inflammatory activities [26]. However, the poor aqueous solubility and low bioavailability of Cur severely limit its clinical applications [27]. To address this issue, various delivery systems, including liposomes [28], micelles [29], emulsions [30], and microcapsules [31], have been explored for the encapsulation of Cur. Among these, liposomes, as phospholipid bilayer vesicles with excellent biocompatibility, can efficiently encapsulate hydrophobic drugs, improving their solubility and stability while enabling sustained release [32], and are now widely regarded as one of the most promising drug carriers [33].

In this study, a Gel/OCS/TGA dual-crosslinked hydrogel was constructed using Gel, OCS, and TGA, followed by the incorporation of Cur@Lipo prepared via microfluidic technology into the hydrogel network, successfully yielding a multifunctional Gel/OCS/TGA/Cur@Lipo composite hydrogel. Specifically, the amino groups on Gel and the aldehyde groups on OCS undergo Schiff-base reactions to form dynamic covalent bonds, while the epoxy groups on TGA react with active groups of Gel and OCS, such as the amino, hydroxyl, and carboxyl groups, yielding a stable covalent crosslinking network. Additionally, the hydroxyl groups generated from ring-opening of the epoxy groups can form hydrogen bonds with the groups of Gel and OCS, synergistically constructing a compact three-dimensional network structure. The mechanical properties, rheological properties and swelling behavior of the Gel/OCS/TGA/Cur@Lipo composite hydrogel were investigated. Additionally, its drug release behavior, antioxidant and antibacterial capacities, as well as in vitro cytotoxicity, were evaluated to determine its suitability for wound dressing applications.

## 2. Results and Discussion

### 2.1. Preparation and Characterization of Cur@Lipo

**Scheme 1:**
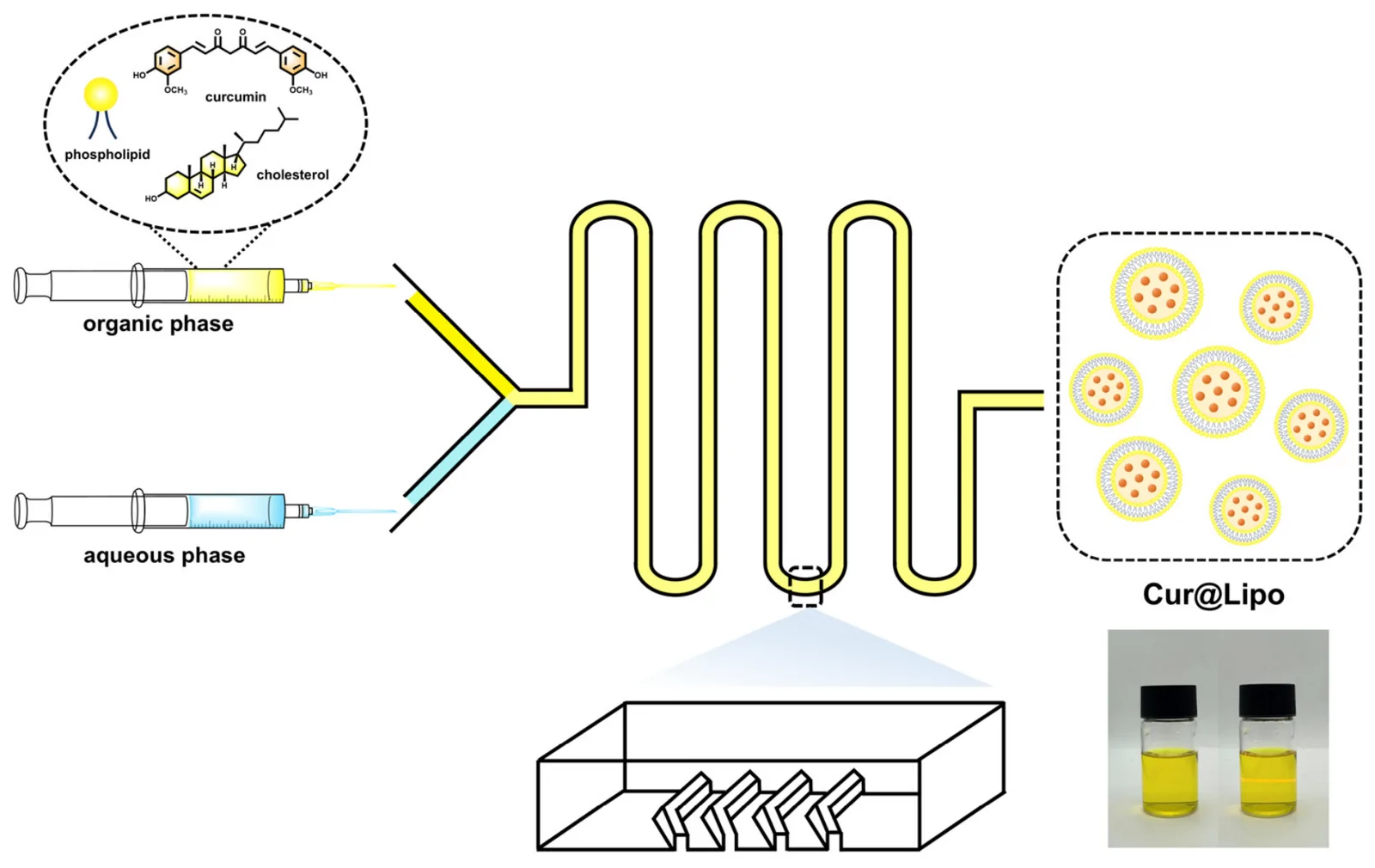
Schematic diagram of the microfluidic preparation of Cur@Lipo.

**Figure 1:**
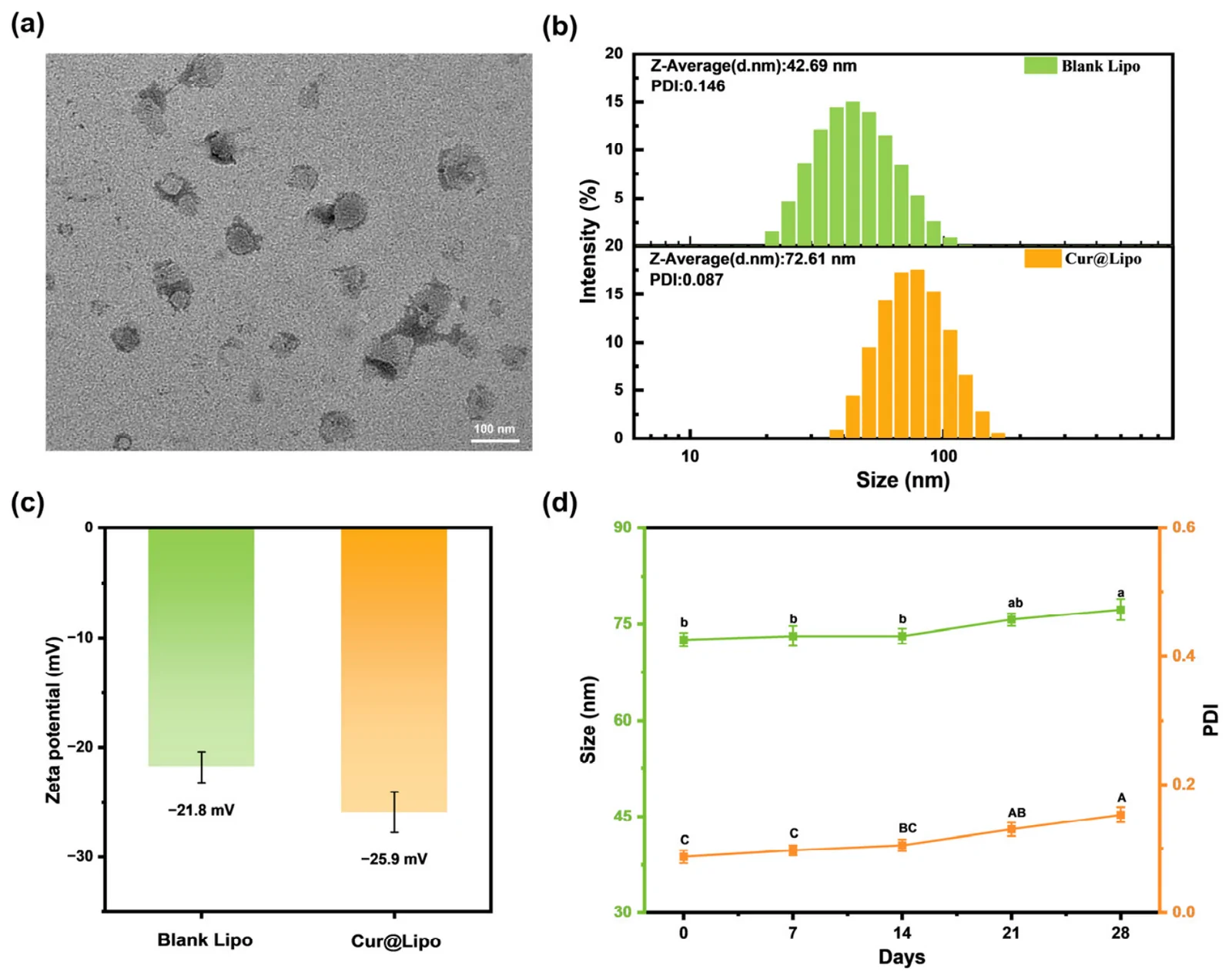
(**a**) TEM images of Cur@Lipo; (**b**) particle size distribution and PDI of Blank Lipo and Cur@Lipo; (**c**) zeta potential measurements of blank Lipo and Cur@Lipo; (**d**) storage stability of Cur@Lipo at 25 °C over 28 days. Different letters on the bars represent statistically significant differences (*p* < 0.05). Lowercase letters denote size, while uppercase letters denote PDI.

The preparation process of Cur@Lipo via microfluidic technology is schematically illustrated in Scheme 1. When a laser beam passed through the resulting Cur@Lipo, a distinct light path was observed, exhibiting a typical Tyndall effect, which indicated that the liposomes were uniformly dispersed in the aqueous phase to form a stable colloidal system. TEM images revealed that Cur@Lipo exhibited a predominantly spherical morphology (Figure 1a). The Blank Lipo exhibited an average diameter of 42.69 nm with a PDI of 0.146, while Cur@Lipo had a larger size of 72.61 nm with a PDI of 0.087 (Figure 1b). The increase in particle size may be attributed to the incorporation of Cur into the liposomal structure [34]. Following Cur loading, the zeta potential of the liposomes decreased from −21.8 mV to −25.9 mV (Figure 1c), which may be attributed to the negatively charged phenolic hydroxyl groups in Cur [35]. The particle size of Cur@Lipo exhibited a slight increase from 72.61 nm to 77.25 nm over 28 days, with a consistently low PDI during the entire storage period (Figure 1d), confirming good storage stability. The UV–Vis absorption spectra revealed that both free Cur and Cur@Lipo exhibited a maximum absorption peak at 425 nm, whereas blank Lipo showed negligible absorption at this wavelength (Figure S1a). These results indicated that the liposomal excipients did not interfere with the UV–Vis absorption of Cur. Based on the calibration curve of the Cur solution (Figure S1b), the encapsulation efficiency (*EE*) and drug loading (*DL*) of Cur@Lipo were calculated to be 81.80 ± 0.14% and 3.51%, respectively. The *EE* was higher than that reported for curcumin liposomes prepared using the conventional thin-film hydration method [36]. This relatively high *EE* and optimal *DL* provided a reliable basis for the subsequent incorporation of Cur@Lipo into the gelatin-based hydrogel.

### 2.2. Synthesis and Characterization of OCS and TGA

**Scheme 2:**
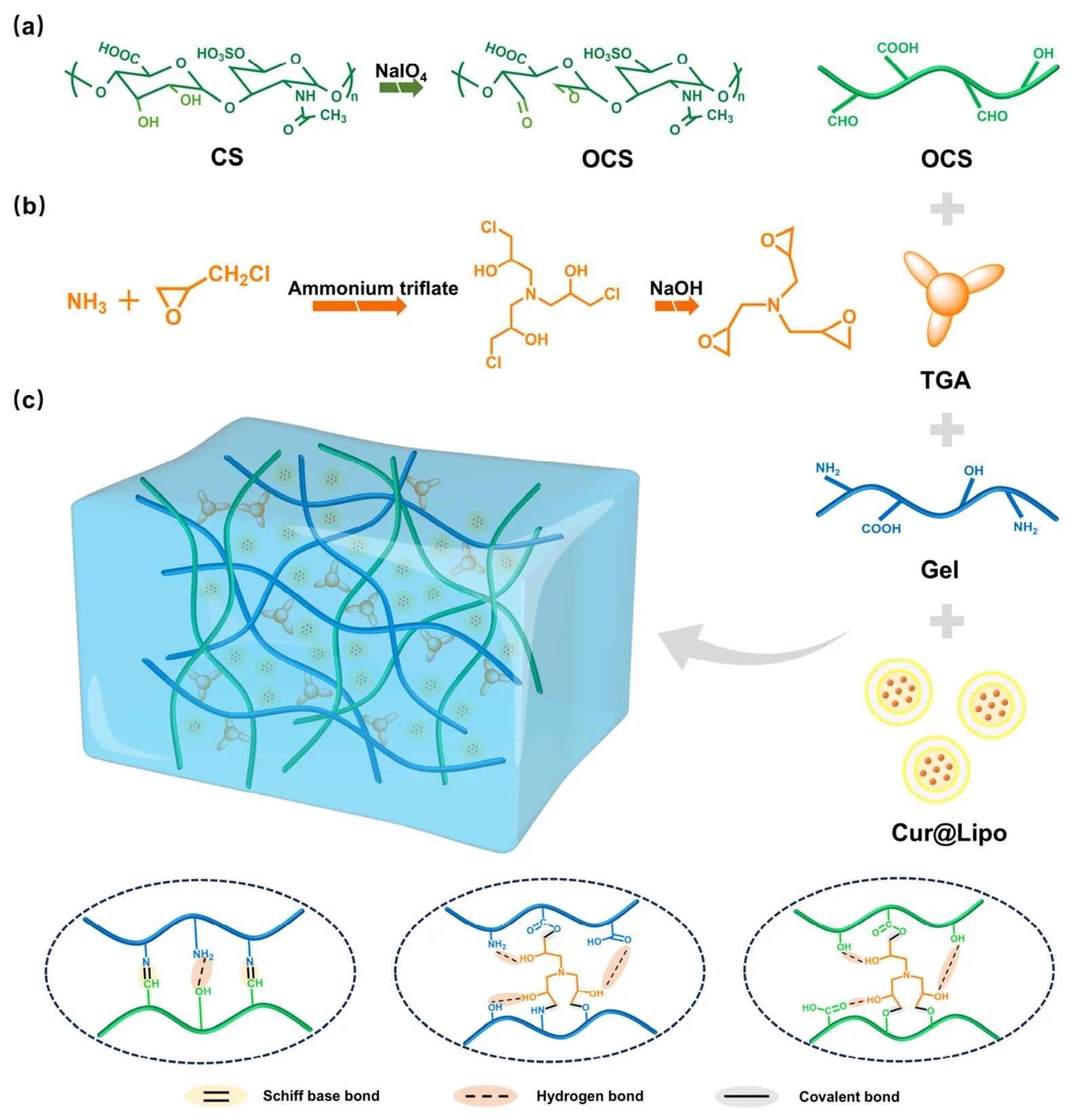
(**a**) Periodate oxidation of CS to OCS; (**b**) synthesis route of TGA; (**c**) schematic illustration of the preparation of multifunctional GOT/Cur@Lipo hydrogel.

**Figure 2:**
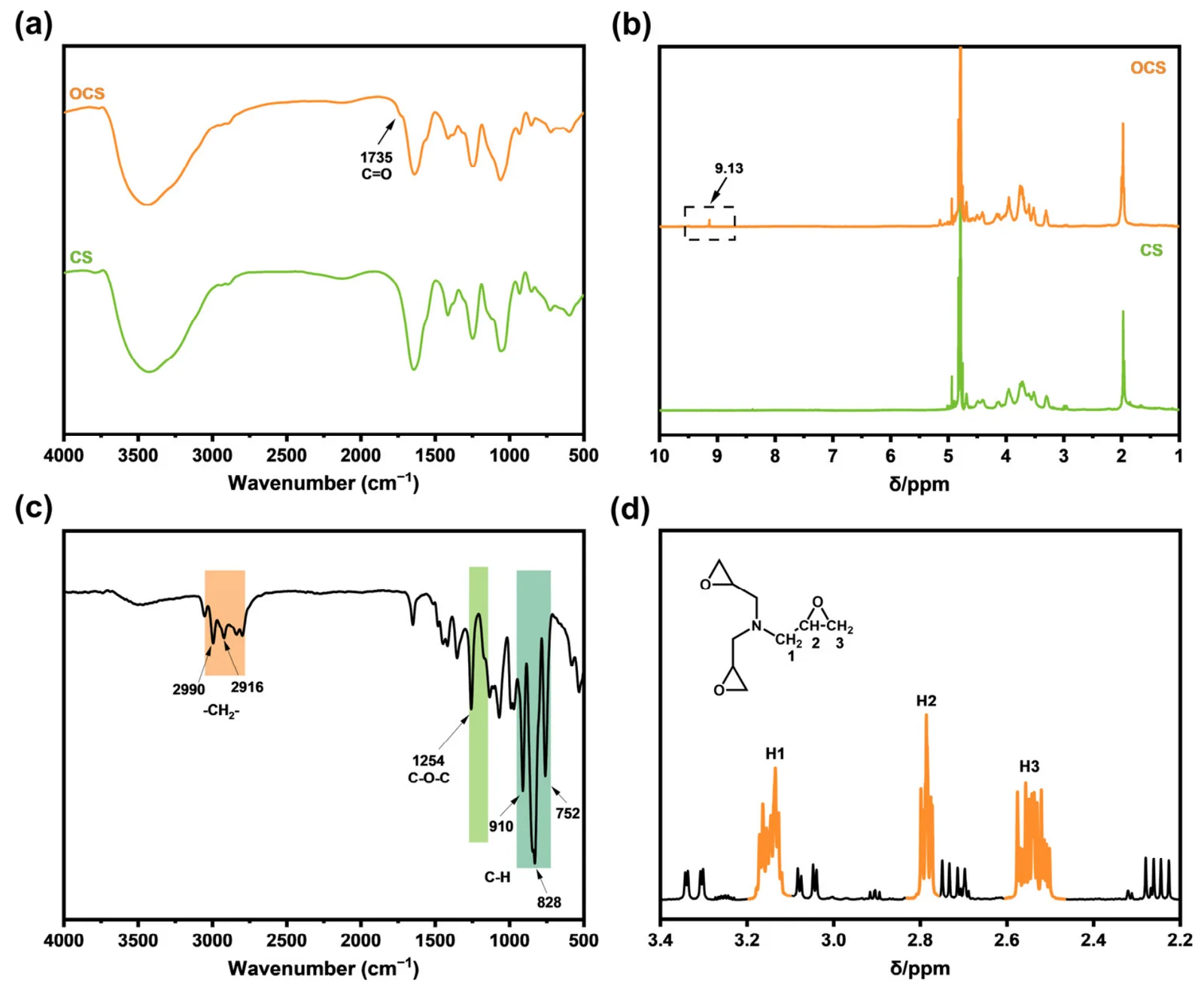
(**a**) FT-IR spectra of CS and OCS; (**b**) ^1^H-NMR spectra of CS and OCS; (**c**) FT-IR spectra of TGA; (**d**) ^1^H-NMR spectrum of TGA.

Sodium periodate (NaIO_4_) selectively oxidized the C–C bonds between vicinal hydroxyl groups in CS, converting them into dialdehyde derivatives, namely OCS (Scheme 2a). The FT-IR spectra of CS and OCS are shown in Figure 2a. In contrast to CS, a new peak appeared at 1735 cm^−1^ in the OCS spectra, assignable to the C=O stretching vibration and confirming the oxidation of hydroxyl to aldehyde groups [37]. In the ^1^H-NMR spectra of CS and OCS, OCS exhibited a characteristic signal at 9.13 ppm, which was assigned to the protons of aldehyde groups [38], whereas no corresponding signal was observed in CS (Figure 2b). The oxidation degree of OCS was determined to be 31.4% using the hydroxylamine hydrochloride method, further confirming the successful oxidation of CS. The introduction of aldehyde groups endowed OCS with enhanced chemical reactivity and provided critical crosslinking sites for subsequent hydrogel network formation.

As illustrated in Scheme 2b, TGA was synthesized through the reaction of epichlorohydrin with ammonia, yielding a product with multiple epoxy functional groups. As shown in Figure 2c, the FT-IR spectrum of TGA exhibited characteristic bands at 2990 and 2916 cm^−1^, assignable to the asymmetric and symmetric stretching vibrations of methylene (–CH_2_–) groups, respectively [39]. The band at 1254 cm^−1^ arose from the C–O–C stretching vibration in epoxy rings [40], while the bands at 910, 828, and 752 cm^−1^ were typical of epoxy moieties [41,42]. Collectively, these bands confirmed the presence of epoxy groups in TGA. In the ^1^H-NMR spectrum of TGA (Figure 2d), the signals at 3.08–3.14 ppm, 2.73–2.77 ppm, and 2.46–2.55 ppm corresponded to the protons of –NCH_2_, –epoxy CH, and –epoxy CH_2_ in TGA. These assignments confirmed that the TGA obtained by this method was a mixture of two diastereomers, in agreement with results of the previous literature [24]. The epoxy value of the resultant TGA was measured to be 1.54 mol/100 g, which was comparable to the theoretical value of 1.62 mol/100 g. Collectively, these results confirmed the successful synthesis of TGA.

### 2.3. Preparation and Characterization of Hydrogels

**Figure 3:**
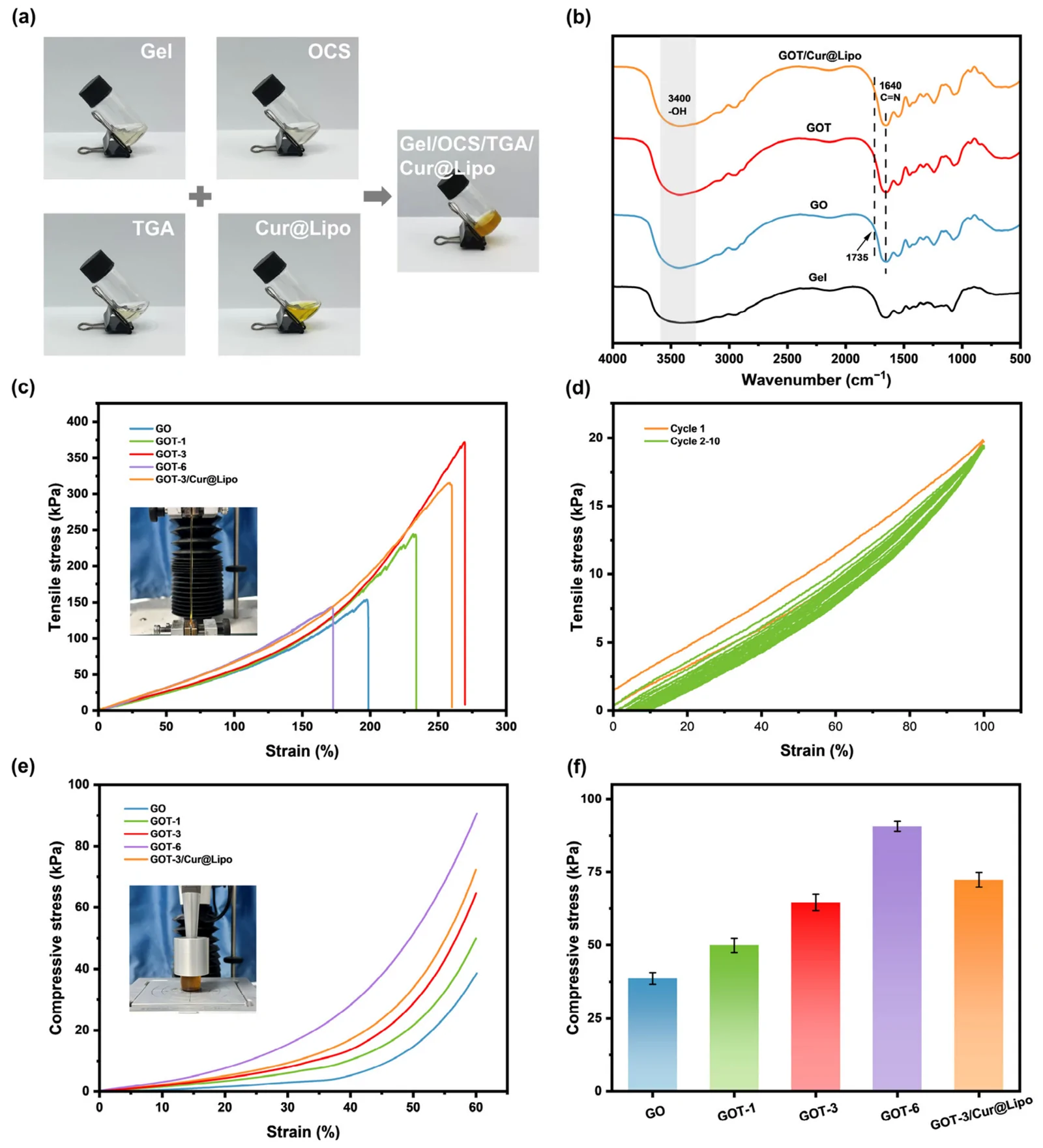
(**a**) Gelling process of GOT/Cur@Lipo hydrogel; (**b**) FT-IR spectra of Gel, GO, GOT and GOT/Cur@Lipo; (**c**) tensile stress–strain curves; (**d**) tensile loading-unloading curves at 100% strain for 10 cycles; (**e**) compressive stress–strain curves; (**f**) compressive stress at 60% strain.

Scheme 2c illustrates the preparation process and crosslinking of the GOT/Cur@Lipo composite hydrogel, featuring a dual-crosslinked network formed through the synergistic integration of dynamic Schiff-base bonds and stable covalent bonds, with Cur@Lipo physically encapsulated within the hydrogel matrix. After TGA addition, the pH of the mixture shifted from 5.0 to 6.0, at which the composite hydrogels were formed. This pH of 6.0 represents a rational compromise among three competing requirements. First, it preserves the stability of OCS, whose aldehyde groups degrade rapidly and irreversibly under alkaline conditions. Second, it permits both the Schiff-base reaction and the TGA-mediated epoxy ring-opening crosslinking to proceed at a workable rate. Third, it is compatible with the encapsulated Cur@Lipo, whose stability is favored under mildly acidic to near-neutral conditions. The sol–gel transition behavior of the hydrogel at 37 °C was evaluated using the vial tilting method (Figure 3a). The FT-IR spectra of Gel, GO, GOT and GOT/Cur@Lipo is shown in Figure 3b. Compared with the hydroxyl band of neat Gel at ~3400 cm^−1^, the corresponding peak of the hydrogels narrowed and intensified. This change likely arose from two factors: the incorporation of OCS, and the generation of additional hydroxyl groups via ring-opening of epoxy moieties in TGA by reactive groups of Gel and OCS. Consequently, hydrogen-bonding interactions within the hydrogel network are reinforced. The characteristic absorption bands of the epoxy moieties at 910, 828, and 752 cm^−1^ were absent in the spectrum of the GOT hydrogel, indicating that the epoxy groups in TGA underwent a ring-opening reaction and supporting the occurrence of the reaction between TGA and Gel/OCS. Furthermore, the disappearance of the aldehyde band at 1735 cm^−1^ and the enhanced absorption band at 1640 cm^−1^ (assigned to the overlap of C=N stretching and the amide I band of Gel) in the composite hydrogel suggested a Schiff-base reaction between aldehyde groups of OCS and amino groups of Gel [43]. The FT-IR spectrum of the GOT/Cur@Lipo composite hydrogel showed no significant difference from that of GOT, which can be attributed to the relatively low incorporation of Cur@Lipo, whose characteristic absorptions (e.g., the alkyl –CH_2_– stretching and phosphate P=O vibrations) were masked by the strong background of the hydrogel matrix.

### 2.4. Mechanical Properties of Hydrogels

The tensile stress–strain curves of the hydrogels are presented in Figure 3c. With an increase in TGA content from 0 to 3 wt%, the tensile strength of the GOT composite hydrogels rose from 153 to 372 kPa, accompanied by an increase in elongation at break from 198% to 269%. This improvement could be attributed to the ring-opening reaction between the epoxy groups of TGA and the reactive moieties in Gel and OCS, which elevated the covalent crosslinking density and effectively reinforced the three-dimensional network structure. However, when the TGA content further increased to 6%, excessive crosslinking imparted greater brittleness to the hydrogel, leading to a drop in tensile strength and elongation at break to 143 kPa and 172%, respectively. Therefore, GOT-3 was selected as the optimal formulation for subsequent experiments. After incorporation of Cur@Lipo, the GOT-3/Cur@Lipo hydrogel showed slightly lower tensile strength (315 kPa) and elongation at break (258%). This reduction was likely due to Cur@Lipo partially occupying the hydrogel network, which may interfere with the formation of covalent crosslinks. The GOT-3/Cur@Lipo hydrogel exhibited a much higher tensile strength than that of a previously reported gelatin/oxidized chondroitin sulfate (Gel/OCS@Ag) hydrogel loaded with silver nanoparticles (117.7 kPa) [44]. The GOT-3/Cur@Lipo hydrogel underwent 10 consecutive tensile loading–unloading cycles at a strain of 100%. A hysteresis loop was observed after the first cycle, indicating energy dissipation within the hydrogel network. From the second to the tenth cycle, the stress–strain curves largely overlapped, and the maximum stress remained relatively stable (Figure 3d), demonstrating good fatigue resistance and recoverability. Figure 3e presents the compressive stress–strain curves of the hydrogel samples. None of the hydrogel samples fractured at a strain of 60%, and the GOT-3/Cur@Lipo hydrogel exhibited a compressive strength of 72 kPa (Figure 3f). For comparison, the previously reported glycidyl methacrylate-modified gelatin/oxidized chondroitin sulfate (Gel-Gym-6/HS-CHO) hydrogel fractured at 60% strain, whereas the Gel-Gym-6/dopamine-functionalized chondroitin sulfate (Gel-Gym-6/HS-Dopa-3) hydrogel exhibited a compressive stress of approximately 10 kPa at the same strain [45].

### 2.5. Morphology of Hydrogels

**Figure 4:**
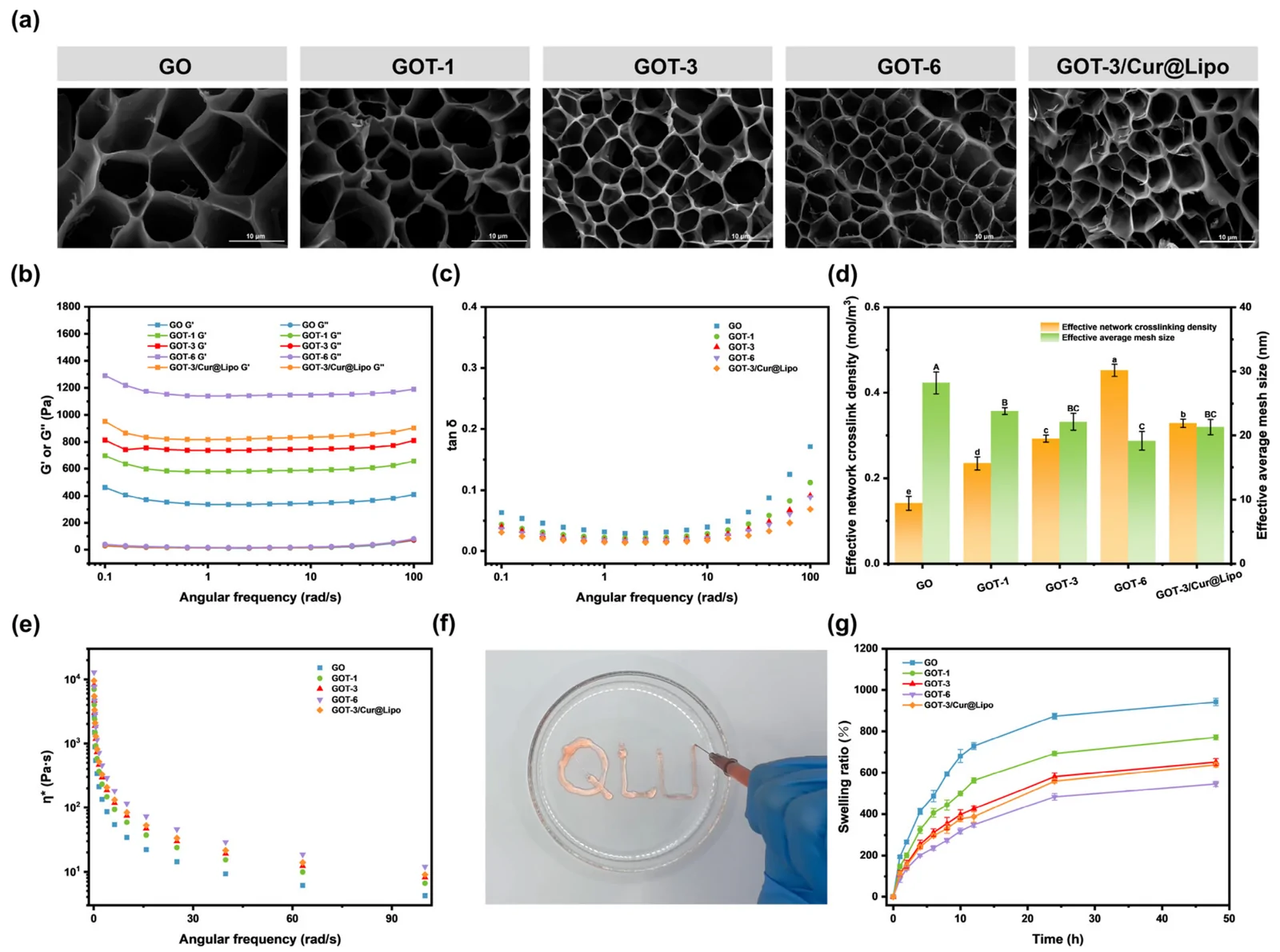
(**a**) SEM images; rheology frequency sweeps of GO, GOT-1, GOT-3, GOT-6 and GOT-3/Cur@Lipo hydrogels: (**b**) *G*′ and *G*″; (**c**) loss factor (*tan δ*); (**d**) effective network crosslink density (*ρ_x_*) and effective average mesh size (*ξ*); (**e**) complex viscosity (η*); (**f**) injectability of GOT-3/Cur@Lipo hydrogel; (**g**) swelling ratio of hydrogels. Different letters on the bars represent statistically significant differences (*p* < 0.05). Uppercase letters denote the effective average mesh size, and lowercase letters denote the effective network crosslink density.

The cross-sectional morphology of the composite hydrogels was observed by SEM (Figure 4a). All hydrogels displayed an interconnected porous network in three dimensions, which may facilitate drug loading and release [46]. The introduction of OCS and TGA promoted the formation of Schiff-base linkages and covalent bonds, thereby increasing the crosslinking density of the hydrogel matrix. Compared with GO, the GOT hydrogel displayed a denser network, and its pore size decreased progressively with rising TGA content. This trend was likely attributable to the reaction between the epoxy groups of TGA and reactive moieties (e.g., amino groups), which generated additional covalent crosslinks. The addition of Cur@Lipo exerted negligible influence on the morphology.

### 2.6. Rheological Properties of Hydrogels

To investigate the internal network structure and mechanical properties of the composite hydrogels, systematic rheological characterizations were performed (Figure 4b–d). Dynamic frequency sweep measurements revealed that the *G*′ remained consistently higher than the *G*″ for all samples (Figure 4b), with *tan δ* values below 0.2 (Figure 4c), indicating viscoelastic solid-like behavior [47]. The results were consistent with other reported Gel/OCS hydrogels [19,48]. As the TGA content increased, *G*′ of the hydrogels rose significantly, suggesting that the incorporation of TGA enhanced the effective crosslinking degree and thereby improved their mechanical properties. Furthermore, higher TGA content led to increased crosslink density and reduced average mesh size of the network (Figure 4d), which was consistent with the SEM observations. Compared with the GOT-3 hydrogel, the incorporation of Cur@Lipo as nanofillers resulted in increased *G*′ and crosslink density. This was likely attributable to the interactions between Cur@Lipo and the hydrogel matrix, which enhanced the structural stability. The relative crosslinking extent of the hydrogels was also evaluated by the ninhydrin assay based on the consumption of free amino groups on gelatin (Figure S2) [49]. The results showed that the relative crosslinking extent increased after TGA addition, and that a higher TGA content led to a higher crosslinking extent. These results were consistent with those obtained from the rheological measurements. The viscosity of all hydrogel samples decreased markedly with increasing angular frequency, confirming shear-thinning behavior—a desirable rheological feature for injectable biomaterials (Figure 4e). As shown in Figure 4f and Movies S1 and S2, the GOT-3/Cur@Lipo hydrogel could be continuously extruded from a syringe to write the letters “QLU”, visually demonstrating its good injectability, which is conducive to adapting to and filling irregular wounds. During the injection process, the reversible disruption of dynamic bonds under high shear stress endows the hydrogel with shear-thinning behavior, allowing it to flow smoothly through the needle. Crucially, once the applied shear force is removed, the rapid self-recovery of the dynamic bonds enables the hydrogel to instantly regain its initial mechanical modulus. This effectively prevents further flow of the material, thereby maintaining the structural integrity post-extrusion.

### 2.7. Swelling Behavior of Hydrogels

An ideal wound dressing should possess good fluid management capability to effectively absorb wound exudate [50]. The swelling ratios of the hydrogels over 48 h are presented in Figure 4g. All hydrogel samples exhibited excellent swelling capacity, with swelling ratios exceeding 480% within the first 24 h. At 48 h, the GOT-3/Cur@Lipo hydrogel reached a swelling ratio of 638%, which was comparable to that of the GOT-3 hydrogel (652%), indicating that the incorporation of Cur@Lipo did not compromise its fluid management capability. Despite such a high water uptake, the hydrogel remained structurally intact without undergoing disintegration. Therefore, it can effectively absorb sufficient wound exudate while maintaining structural stability. The mass loss of GOT-3/Cur@Lipo after 48 h of swelling was 15.39 ± 1.41%, which is mainly attributable to the dissolution of uncrosslinked gelatin and OCS, the release of Cur@Lipo, and, to a lesser extent, the leaching of unreacted residual TGA.

### 2.8. Adhesion and Self-Healing Properties of Hydrogels

**Figure 5:**
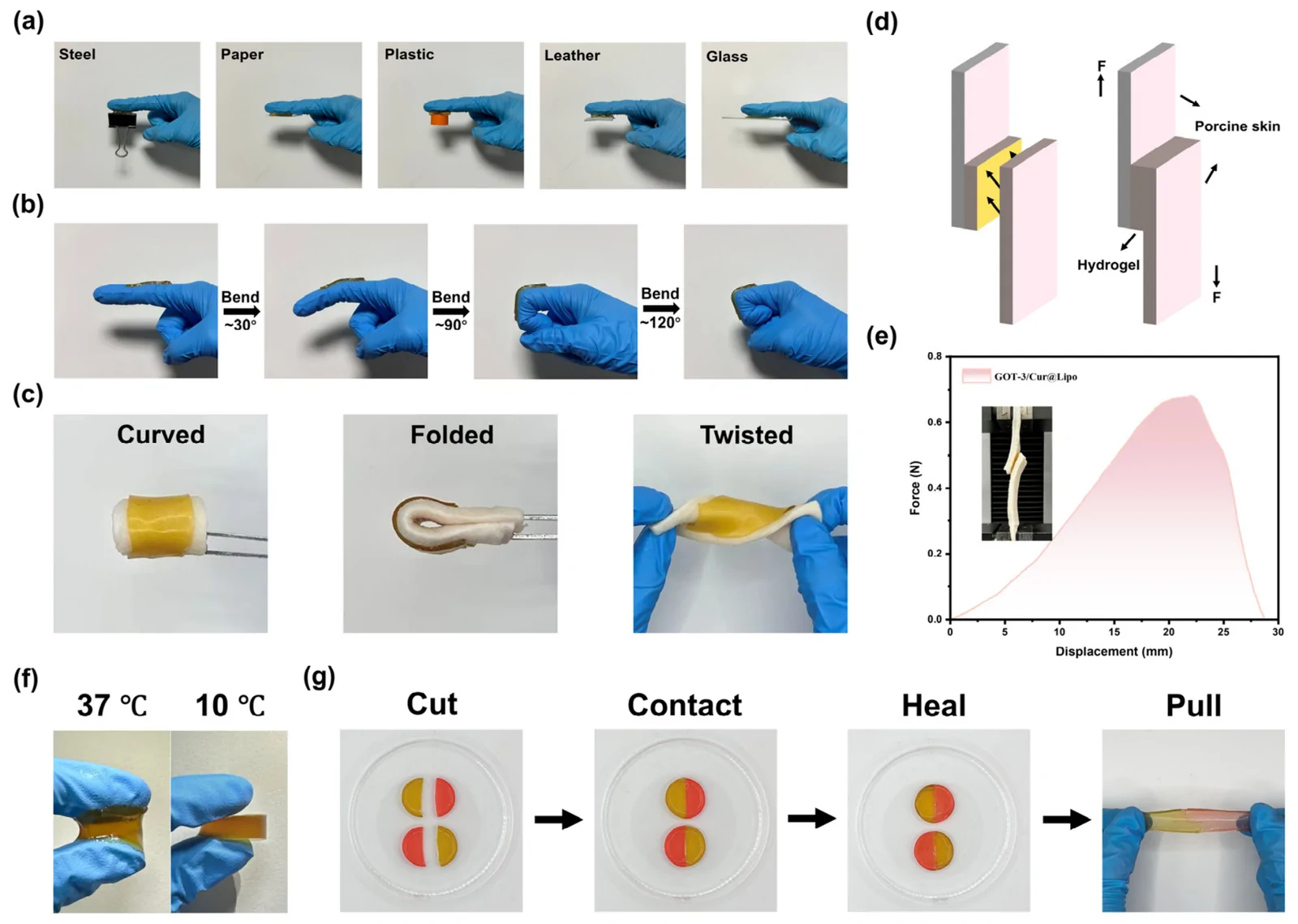
(**a**) Adhesion photographs of GOT-3/Cur@Lipo hydrogel to different materials; (**b**) photographs of flexibility and skin conformability of GOT-3/Cur@Lipo hydrogel when applied to a finger during bending; (**c**) adhesion photographs of GOT-3/Cur@Lipo hydrogel to porcine skin; (**d**) schematic diagram of the lap shear test using porcine skin; (**e**) adhesion strength curve of GOT-3/Cur@Lipo hydrogel; (**f**) adhesive behavior of the GOT-3/Cur@Lipo hydrogel at 37 °C and 10 °C; (**g**) photographs of self-healing process of the GOT-3/Cur@Lipo hydrogel.

The adhesive properties of GOT-3/Cur@Lipo hydrogel were qualitatively evaluated, as shown in Figure 5a. The hydrogel exhibited good adhesion to a variety of materials, including steel, paper, plastic, leather, glass, and nitrile gloves. When attached to a finger joint, the hydrogel remained firmly in place during free joint bending, demonstrating good dynamic adaptability (Figure 5b). To mimic the practical application of a wound dressing, GOT-3/Cur@Lipo was placed on the surface of porcine skin. After repeated folding and twisting, the hydrogel remained closely conformed to the skin without detachment (Figure 5c), indicating good tissue adhesive capability. This adhesive capability is mainly attributed to the abundant active functional groups in Gel, OCS, and TGA after epoxy ring-opening, which can form hydrogen bonds, electrostatic interactions and other physical interactions with active groups on the material surface, thereby enabling stable and durable interfacial adhesion. A lap-shear test was further performed to quantitatively evaluate the adhesive properties of the hydrogel (Figure 5d). The adhesive strength of the GOT-3/Cur@Lipo composite hydrogel reached 6.86 kPa (Figure 5e), surpassing that of a commercial fibrin glue (5 kPa) [51], rhein- and tetracaine-loaded gelatin/C-phycocyanin/silk fibroin hydrogel (3.97 kPa) [52] and oxidized sodium alginate/quaternized carboxymethyl chitosan hydrogel (3.08 kPa) [53]. The hydrogel adhered strongly to skin at 37 °C, but its adhesive capability diminished when the temperature was lowered to 10 °C, demonstrating thermoresponsive reversible adhesion (Figure 5f). This phenomenon may be attributed to the regulation of hydrogel chain mobility and interfacial contact state by temperature variations [48]. This characteristic enables the hydrogel to remain stably attached to the wound under physiological temperature while allowing for on-demand and easy detachment at lower temperatures, thereby facilitating dressing replacement and minimizing secondary damage to the wound.

Benefiting from the reversible dynamic Schiff-base bonds, the GOT-3/Cur@Lipo hydrogel could achieve self-healing without any external stimulation. As shown in Figure 5g, the GOT-3/Cur@Lipo was evenly split into two pieces and kept in close contact at 37 °C for 1 h. The healed hydrogel could be stretched without fracture, indicating its self-healing capability. The *G*′ and *G*″ of the hydrogel before and after healing are presented in Figure S2, yielding a healing efficiency of 92.02%. When the fractured surfaces of the hydrogel were brought back into contact, the polymer chains at the interface underwent diffusion and rearrangement, allowing the Schiff-base bonds to reform and thereby facilitating dynamic self-healing [54].

### 2.9. Antibacterial and Antioxidant Activities of Hydrogels

**Figure 6:**
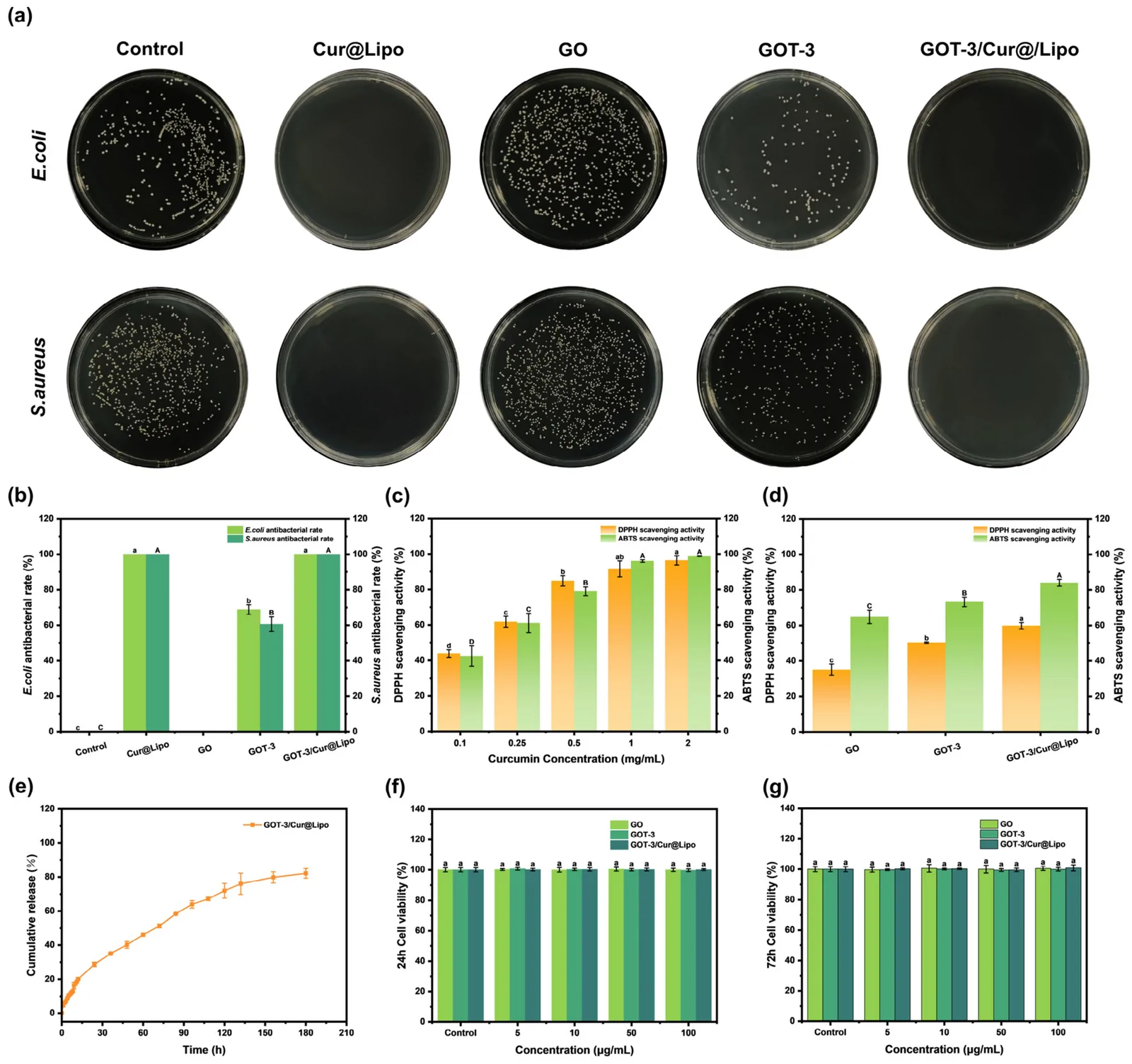
(**a**) Photographs of antibacterial activity and (**b**) antibacterial rate of Cur@Lipo, GO, GOT-3 and GOT-3/Cur@Lipo hydrogels against *E. coli* and *S. aureus*; (**c**) antioxidant activities of Cur@Lipo; (**d**) antioxidant activities GO, GOT-3, GOT-3/Cur@Lipo hydrogels; (**e**) cumulative release of Cur from GOT-3/Cur@Lipo hydrogels; (**f**) relative cell viability after 24 h; (**g**) relative cell viability after 72 h. Different letters on the bars represent statistically significant differences (*p* < 0.05). In Figure 6b, uppercase letters denote antibacterial rate of *E. coli*, and lowercase letters denote that of *S. aureus*; in Figure 6c,d, uppercase letters denote ABTS scavenging rate, and lowercase letters denote DPPH scavenging rate.

Effective antibacterial activity can reduce the risk of wound infection and promote wound healing [55]. The antibacterial activities of Cur@Lipo and the composite hydrogels were evaluated using a plate-counting assay. Figure 6a presents the colony growth of *E. coli* and *S. aureus* after co-incubation with the various samples. Cur@Lipo alone achieved antibacterial rates exceeding 99.99% (corresponding to a ≥4-log reduction) against both strains (Figure 6b), demonstrating excellent antibacterial activity. Thus, Cur@Lipo can serve as an effective antibacterial component in hydrogel wound dressings. In contrast, the GO hydrogel exhibited no antibacterial effect against either strain. Upon introduction of TGA, the GOT-3 hydrogel showed bacterial reduction rates of 68.78% and 60.58% against *E. coli* and *S. aureus*, respectively. This activity was ascribed to the cationic nature of the tertiary amine groups in TGA, which interacted electrostatically with negatively charged bacterial membranes. After further loading with Cur@Lipo, the GOT-3/Cur@Lipo hydrogel displayed excellent antibacterial performance, with reduction rates exceeding 99.99% (corresponding to a ≥4-log reduction) for both strains, attributable to the combined effects of TGA and Cur@Lipo.

Excessive reactive oxygen species (ROS) at the wound site can induce severe oxidative stress and impair the wound healing process, highlighting the importance of antioxidant activity in hydrogel dressings [56]. The antioxidant activities of Cur@Lipo and the composite hydrogels were evaluated using DPPH and ABTS radical scavenging assays. At a Cur@Lipo concentration of 0.5 mg/mL, the DPPH and ABTS radical scavenging rates both exceeded 80%, demonstrating strong antioxidant activity (Figure 6c). A higher Cur@Lipo concentration resulted in an increased radical scavenging rate, which can be attributed to the higher Cur concentration. As a polyphenolic compound, Cur exhibits antioxidant activity, and its phenolic hydroxyl groups can effectively scavenge free radicals and excessive ROS [57]. The DPPH/ABTS radical scavenging rates of free Cur and Cur@Lipo were measured at equivalent Cur concentrations, and the results are shown in Figure S4. Both samples exhibited high and comparable scavenging rates, indicating that encapsulation of Cur in liposomes did not compromise its antioxidant activity. Figure 6d shows the antioxidant activities of the hydrogel samples. The GOT-3/Cur@Lipo hydrogel exhibited DPPH and ABTS radical scavenging rates of 59.8 ± 1.83% and 83.98 ± 1.91%, respectively, which were higher than those of the GO hydrogel (35.05 ± 3.18% and 64.77 ± 3.77%, respectively) and the GOT-3 hydrogel (50.20 ± 0.37% and 73.28 ± 2.76%, respectively). The antioxidant activity of the GO hydrogel originated from the intrinsic radical-scavenging capacity of Gel and OCS. After TGA incorporation, GOT-3 showed elevated scavenging rates, likely due to the hydroxyl groups generated by epoxy ring-opening and the tertiary amine moieties of TGA. The GOT-3/Cur@Lipo hydrogel exhibited the highest activity, which was mainly attributed to Cur, with additional contributions from the Gel/OCS matrix and TGA-derived groups.

### 2.10. In Vitro Drug Release

Figure 6e presents the in vitro Cur release profile of the GOT-3/Cur and GOT-3/Cur@Lipo composite hydrogel under simulated physiological conditions (PBS, pH 7.4, with 1% Tween-80). The GOT-3/Cur@Lipo composite hydrogel exhibited sustained Cur release for up to 180 h. During the initial 0–12 h period, the cumulative release was 20.17%. Thereafter, the release profile showed a gradual and sustained increase from 24 to 180 h, reaching a cumulative release of 82.11%. For comparison, the Cur release behavior of GOT-3/Cur—a hydrogel containing the same concentration of free Cur instead of Cur@Lipo—was also examined (Figure S5), which exhibited 36.42% release within 12 h and reached 80.48% cumulative release after 84 h. GOT-3/Cur@Lipo effectively suppressed the initial burst and enabled sustained curcumin delivery. This sustained release behavior can be attributed to the encapsulation of Cur within liposomes: the highly hydrophobic Cur was confined within the phospholipid bilayer of the liposomes, thereby hindering its direct diffusion into the aqueous medium. In addition, the hydrogel matrix has also been reported to slow down drug release [58].

### 2.11. In Vitro Cytotoxicity

Good cytocompatibility is a prerequisite for biomaterials intended for in vivo applications. Figure 6f,g shows the cell viability of HUVECs after co-culture with different composite hydrogels for 24 and 72 h. All samples maintained high cell viability (>98%) after co-culture for 24 and 72 h, and no concentration-dependent cytotoxicity was observed. Therefore, all hydrogel samples exhibited good in vitro cytocompatibility.

## 3. Materials and Methods

### 3.1. Materials

Gel (182 Bloom) was supplied by Shandong Jiele Biotechnology Co., Ltd. (Jinan, China). CS was purchased from Shanghai Bide Pharmaceutical Technology Co., Ltd. (Shanghai, China). Sodium periodate, ammonium triflate, Cur, Epichlorohydrin, ammonium hydroxide, soy lecithin and cholesterol were purchased from Sinopharm Chemical Reagent Co., Ltd. (Shanghai, China).

### 3.2. Preparation of Cur@Lipo

Cur@Lipo were prepared using microfluidic technology according to the literature [59]. The specific steps were as follows: 90 mg of soy lecithin, 22.5 mg of cholesterol, and 5 mg of Cur were solubilized in anhydrous ethanol as the organic phase; deionized water was used as the aqueous phase. The two phases were drawn into syringes and then pumped into the inlets of the microfluidic chip, which features serpentine micromixing channels equipped with protruding herringbone structures. The microfluidic operating parameters were set to a total flow rate of 0.5 mL/min and an aqueous-to-organic flow rate ratio of 3:1. After the flow rate was steady, the Cur@Lipo was collected at the outlet. Blank liposomes (Blank Lipo) were prepared using the same conditions as Cur@Lipo, except without Cur. The as-prepared samples were preserved at 25 °C in the absence of light before testing.

### 3.3. Preparation of Gel/OCS/TGA/Cur@Lipo Composite Hydrogel

#### 3.3.1. Preparation of OCS

CS (3 g) was solubilized in 100 mL of deionized water, and then 0.5 mol/L sodium periodate was added as the oxidant. The mixture was magnetically stirred at 500 rpm under dark conditions at 25 °C for 8 h, after which the reaction was quenched with 2 mL of ethylene glycol and stirred for another 1 h at room temperature. The resulting product was dialyzed (MWCO: 3.5 kDa) against deionized water for 72 h, lyophilized, and preserved at −20 °C. The oxidation degree of the obtained OCS was determined via the hydroxylamine hydrochloride method [60].

#### 3.3.2. Preparation of TGA

TGA was synthesized according to a previously reported method [24]. Briefly, ammonium hydroxide was added dropwise to a solution of epichlorohydrin, isopropanol, and ammonium triflate, and the resulting mixture was then magnetically stirred at 50–55 °C for 48 h. The solvent and excess epichlorohydrin were removed by rotary evaporation to obtain a colorless viscous liquid. Then, 50% (*w*/*w*) NaOH was added, stirred at 18–22 °C for 2 h, and diluted with ice-cold water to dissolve the formed NaCl. The organic layer was collected and subjected to vacuum distillation to yield TGA. The epoxy value of TGA was determined in accordance with the national standard method (GB/T 1677-2023) [61].

#### 3.3.3. Preparation of Gel/OCS/TGA/Cur@Lipo Composite Hydrogel

Gel and OCS were dissolved in deionized water to 30% and 10% (*w*/*v*), respectively, and mixed at 37 °C at a 1:1 (*v*/*v*) ratio to obtain GO. TGA was then added at 1%, 3%, or 6% (*w*/*w* based on Gel) to yield GOT-1, GOT-3, and GOT-6, respectively. After stirring for 15 min, each mixture was poured into PTFE molds and allowed to crosslink at 37 °C. The optimal GOT-3 was further loaded with Cur@Lipo to obtain GOT-3/Cur@Lipo. The detailed compositions of the GOT/Cur@Lipo hydrogels are shown in Table S1.

### 3.4. Transmission Electron Microscope (TEM) Observation of Cur@Lipo

The morphology of Cur@Lipo was observed using a JEM 2100 TEM (JEOL Ltd., Akishima, Japan) at an accelerating voltage of 200 kV.

### 3.5. Particle Size Distribution, Polydispersity Index (PDI) and Zeta Potential Measurement

The particle size distribution, PDI, and zeta potential of Blank Lipo and Cur@Lipo were determined using a Malvern Zetasizer (ZEN3600, Malvern Instruments Ltd., Malvern, UK) at 25 °C.

### 3.6. Storage Stability Evaluation of Cur@Lipo

To assess the storage stability, the particle size and PDI of Cur@Lipo were measured on days 0, 7, 14, 21, and 28 during storage at 25 °C in the absence of light.

### 3.7. Encapsulation Efficiency (EE) and Drug Loading (DL) of Cur@Lipo

To determine the maximum absorption wavelength of Cur, UV-Vis spectra of Cur, Blank Lipo, and Cur@Lipo were acquired on a UV-2600 spectrophotometer (Shimadzu Corp., Kyoto, Japan) across the 200–800 nm range. A standard curve was then established at this wavelength using Cur solutions of varying concentrations. The *EE* and *DL* of Cur@Lipo were determined by membrane filtration method [62]. Briefly, 1 mL of the filtered and unfiltered Cur@Lipo was diluted to 20 mL with methanol and sonicated for 30 min. The absorbance was measured at 425 nm to calculate the amount of encapsulated curcumin and total amount of curcumin. The *EE* and *DL* of Cur@Lipo was calculated using the following formula:

(1) $$EE\left(\%\right)=\frac{W_{en}}{W_{total}}\times 100\%\;$$

(2) $$DL\left(\%\right)=\frac{W_{en}}{W_{en}+W_{lipo}}\times 100\%$$

where $W_{en}$ is the amount of Cur entrapped in the liposomes, $W_{total}$ is the total amount of Cur initially added, and $W_{lipo}$ is the total amount of soy lecithin and cholesterol.

### 3.8. Fourier Transform Infrared (FT-IR) Spectra

The FT-IR spectra of CS, OCS, TGA, GOT, and GOT/Cur@Lipo composite hydrogel were recorded using a FT-IR spectrometer (IRAffinity-1S, Shimadzu Corp., Kyoto, Japan). Each sample underwent 32 scans with a resolution of 4 cm^−1^, covering a spectral range from 4000 to 500 cm^−1^.

### 3.9. Nuclear Magnetic Resonance (NMR) Spectra

The ^1^H-NMR spectra of the CS, OCS and TGA were recorded using a 400 MHz NMR spectrometer (JNM-ECZL400S, JEOL Ltd., Akishima, Japan). CS and OCS were dissolved in D_2_O, while TGA was dissolved in deuterated DMSO, at a concentration of 10 mg/mL prior to measurement.

### 3.10. Mechanical Properties of Hydrogels

A texture analyzer (TA.XT Plus, Stable Micro Systems Ltd., Godalming, UK) was employed to assess the mechanical properties of the hydrogels. Dumbbell-shaped specimens (length × width × height = 50 × 8.5 × 2 mm) were used for tensile tests at a constant rate of 20 mm/min, and loading–unloading cycles were performed at 100% strain for 10 cycles. Compression tests were conducted on cylindrical specimens (diameter × height = 20 × 10 mm) at a rate of 5 mm/min.

### 3.11. Scanning Electron Microscopy (SEM)

An EM 30 Plus+ SEM (COXEM Co., Ltd., Daejeon, Republic of Korea) operating at 20 kV was employed for cross-sectional observation of the freeze-dried hydrogels, which were previously sputter-coated with gold.

### 3.12. Rheological Properties of the Hydrogels

A rheometer (ARES-G2, Waters Corp., Milford, MA, USA) was employed to assess the rheological behavior of the hydrogels. Oscillatory frequency sweeps (0.1–100 rad/s, 1% strain) provided the storage modulus (*G*′), loss modulus (*G*″), and complex viscosity (η*). From these data, *tan δ*, effective network crosslink density (*ρ_x_*) and effective average mesh size (*ξ*) were calculated according to the following formulas [63]:

(3) $$Tan\;\delta =G^{\prime \prime }/G^{\prime }$$

(4) $$\rho_{X}=G/RT\;$$

where G is the average storage modulus over the frequency range (Pa), R is the universal gas constant (8.314 Pa·m^3^/(mol·K)), and T is the absolute temperature (K).

(5) $$\xi =\sqrt[{3}]{\frac{6}{\pi \rho_{x}N_{A}}}$$

where $N_{A}$ is the Avogadro constant (6.02 × 10^23^).

### 3.13. Swelling Behavior of the Hydrogels

Prior to testing, the hydrogels were freeze-dried for 48 h until constant weight, and their initial mass (*W_i_*) was recorded. The samples were then submerged in phosphate-buffered saline (PBS, pH 7.4) at 37 °C. At predetermined time intervals (1, 2, 4, 6, 8, 10, 12, 24 and 48 h), individual specimens were retrieved, carefully patted dry with filter paper to eliminate surface moisture and reweighed to determine the swollen mass (*W_s_*). After 48 h of swelling, the sample was freeze-dried again to obtain the final mass (*W*_48_). The swelling ratio was determined using the following formula [64]:

(6) $$Swelling\;ratio=\left(W_{s}-W_{i}\right)/W_{i}\times 100\%$$

The mass loss of was determined using the following formula:

(7) $$Mass\;loss=\left(W_{i}-W_{48}\right)/W_{i}\times 100\%\;$$

### 3.14. Adhesive Properties of the Hydrogels

The adhesive properties of the hydrogels were evaluated using a lap-shear test. Briefly, fresh porcine skin was defatted and cut into 40 × 10 mm strips. A 10 × 10 mm hydrogel sheet was placed between two pieces of porcine skin and allowed to stabilize at 37 °C for 30 min. The adhesive strength was then measured using a universal testing machine (Instron 5943, Instron Corp., Norwood, MA, USA) at a rate of 20 mm/min and calculated according to the following formula:

(8) P=F/A

where P is the adhesive strength, F is the maximum tensile force (N), and A is the adhesion area (mm^2^).

### 3.15. Antibacterial Performance

The growth inhibition of *E. coli* and *S. aureus* by Cur@Lipo and the hydrogels was assessed by the plate count method. Briefly, the hydrogel (0.1 g/mL) were incubated with bacteria (1 × 10^6^ CFU/mL) for 2 h; the control was prepared under identical conditions without the addition of any sample. Then, aliquots of each mixture (100 µL) were evenly plated on agar and incubated at 37 °C for 12–18 h. The colonies were counted and the antibacterial rate was computed by the following formula:

(9) $$Antibacterial\;rate=\left(C_{0}-C_{t}\right)/C_{0}\times 100\%$$

where $C_{0}$ refers to the numbers of colonies for the control group, while $C_{t}$ refers to those of the experimental group.

### 3.16. Antioxidant Activity

Cur@Lipo solutions at concentrations of 0.1, 0.25, 0.5, 1, and 2 mg/mL were prepared. Hydrogel samples (GO, GOT-3, and GOT-3/Cur@Lipo, 200 mg each in the hydrated state) were incubated in 10 mL deionized water at 37 °C for 12 h to obtain extracts at 20 mg/mL. Then, 1 mL of the sample solution was mixed with an equal volume of DPPH ethanol solution (0.1 mM) and kept in the absence of light for 30 min, after which the absorbance at 517 nm was measured. The DPPH radical scavenging activity was calculated according to the following formula:

(10) $$DPPH\;radical\;scavenging\;activity=\left(1-\frac{A_{1}-A_{2}}{A_{0}}\right)\times 100\%$$

where $A_{0}$, $A_{1}$, and $A_{2}$ represent the absorbance of the control group (DPPH with deionized water), the experimental group (DPPH with sample solution), and the blank group (ethanol with sample solution), respectively.

A quantity of 1 mL of sample solution was mixed with an equal volume of ABTS solution and placed in the dark for 6 min, after which the absorbance at 734 nm was recorded. The ABTS radical scavenging activity was calculated according to the following formula:

(11) $$ABTS\;radical\;scavenging\;rate=\left(1-\frac{A_{1}-A_{2}}{A_{0}}\right)\times 100\%\;$$

where $A_{0}$, $A_{1}$, and $A_{2}$ represent the absorbance of the control group (ABTS with deionized water), the experimental group (ABTS with sample solution), and the blank group (deionized water with sample solution), respectively.

### 3.17. In Vitro Drug Release

The in vitro Cur release from the GOT-3/Cur and GOT-3/Cur@Lipo hydrogel was monitored in PBS (pH 7.4) with 1% (*w*/*v*) Tween-80 at 37 °C. The hydrogel was placed in 50 mL of this medium and agitated under dark conditions. At designated intervals, 3 mL aliquots were taken out and exchanged with an equal volume of fresh buffer. The released Cur was quantified by UV–Vis spectroscopy at 425 nm using a standard curve. The cumulative release rate was calculated using the following equation:

(12) $$Cumulative\;release=\frac{C_{n}V+\sum_{i=1}^{i=n-1}C_{i}V_{i}}{m}\times 100\%\;$$

where V and $V_{i}$ are the total volume of the release medium and the sampling volume, $C_{n}$ and $C_{i}$ are the Cur concentration at the nth and ith sampling time, and m is the total mass of Cur loaded in the hydrogel.

### 3.18. In Vitro Cytotoxicity

Cytocompatibility of the hydrogels toward human umbilical vein endothelial cells (HUVECs) was examined by the MTT assay. Extract solutions with an initial concentration of 0.1 g/mL were prepared by incubating the samples in complete medium at 37 °C for 24 h. HUVECs (log phase) were seeded in 96-well plates (6 × 10^3^/well) and treated with 100 μL of the diluted extracts. Fresh medium and cell-free medium were used as the control and blank, respectively. After 24 and 72 h of incubation at 37 °C (5% CO_2_), the culture medium was removed, and each well was washed three times with PBS. Subsequently, 100 μL of fresh medium containing 0.5 mg/mL MTT was added to each well and incubated for an additional 4 h. The supernatant was carefully discarded, and 100 μL of dimethyl sulfoxide (DMSO) was added to each well to dissolve the formed crystals. After gentle shaking for 10 min, the OD at 570 nm was determined using a microplate reader (Spark 10M, Tecan, Männedorf, Switzerland). The relative cell viability was calculated according to the following formula:

(13) $$Cell\;viability=\frac{{OD}_{s}-{OD}_{b}}{{OD}_{c}{-OD}_{b}}\times 100\%\;$$

where $OD_{s}$, $OD_{b}$, and $OD_{c}$ refer to the absorbance of the sample group, the blank group, and the control group, respectively.

### 3.19. Statistical Analysis

All experiments were performed in triplicate, and data are presented as mean ± SD. Statistical analysis and data visualization were conducted using Origin 2024 software, with statistical significance defined as *p* < 0.05.

## 4. Conclusions

In this work, we designed a gelatin-based composite hydrogel (GOT/Cur@Lipo) featuring a dual-crosslinked network and liposome-encapsulated curcumin as a multifunctional wound dressing. The Schiff-base linkages between Gel and OCS endowed the hydrogel with self-healing capability and injectability, while the covalent crosslinking introduced by TGA significantly reinforced the mechanical performance, achieving a tensile strength of 315 kPa, strain of 258%, and compressive stress of 72 kPa at 60% strain. The incorporation of Cur@Lipo, prepared via microfluidic technology, not only imparted antioxidant (DPPH and ABTS radical scavenging rates of 59.8% and 83.98%, respectively) and antibacterial activities (with bacterial inhibition rates ~100% against *E. coli* and *S. aureus*), but also ensured a controlled drug release profile over 180 h without compromising the hydrogel’s cytocompatibility. Overall, we provide an effective strategy to integrate outstanding mechanical properties and multiple biofunctions into one unified platform, which successfully overcomes the intrinsic limitations of conventional gelatin hydrogels. The GOT/Cur@Lipo hydrogel shows potential as a wound dressing candidate, and future in vivo full-thickness wound healing evaluations will be conducted to further assess its clinical translation.

## Data Availability

Data is contained within the article or Supplementary Materials. Further inquiries can be directed to the corresponding authors.

## Supplementary Materials

The following supporting information can be downloaded at: https://www.mdpi.com/article/10.3390/molecules31213868/s1, Table S1. Compositions of the prepared hydrogels. Figure. S1. (a) UV-Vis absorption spectra of Cur, Blank Lipo, and Cur@Lipo; (b) standard curve of curcumin at 425 nm. Figure S2. (a) Standard curve of glycine solutions; (b) relative crosslinking extent of GO, GOT-1, GOT-3 and GOT-6 hydrogels. Figure S3. G′ and G″ of the GOT-3/Cur@Lipo hydrogel before and after self-healing. Figure S4. Antioxidant activities of free Cur and Cur@Lipo at equivalent curcumin concentrations. Different letters on the bars represent statistically significant differences (*p* < 0.05). Figure S5. Cumulative release of curcumin from GOT-3/Cur hydrogels. Movie S1. The injectability of GOT-3/Cur@Lipo hydrogel. Movie S2. GOT-3/Cur@Lipo hydrogel continuously writes the letter “QLU” through a syringe.
