# Supplementary material for: Dual-Crosslinked Gelatin/Chondroitin Sulfate Hydrogel Loaded with Curcumin Liposomes for Multifunctional Wound Dressing
Source: Molecules. 2026 Oct 27;31(21):3868. doi: 10.3390/molecules31213868 (PMC13765280; doi:10.3390/molecules31213868)
Supplement: Supplementary file 1 [file molecules-31-03868-s001.zip › Supplementary Materials.pdf]

Supplementary Materials

Dual-Crosslinked Gelatin/Chondroitin Sulfate Hydrogel Loaded with Curcumin Liposomes for Multifunctional Wound Dressing

Jiatong Han [1], Pengfei Qiao [1], Daijie Wang [2], Hao Li [1], Liqiang Jin [1,*] and Qinghua Xu [3,*]

[1] Faculty of Light Industry, Qilu University of Technology (Shandong Academy of Sciences), Jinan 250353, China; 13165257918@163.com (J.H.); q1191493231@163.com (P.Q.); lihao@qlu.edu.cn (H.L.)

[2] Food Resources Development and Health Product Creation International Joint Laboratory, Innovation Center for High-Value Development and Utilization of Bio-Health Food Technology of Shandong Province, Heze Branch of Qilu University of Technology (Shandong Academy of Sciences), Heze 274000, China; wangdaijie@qlu.edu.cn

[3] State Key Laboratory of Green Papermaking and Resource Recycling, Qilu University of Technology (Shandong Academy of Sciences), Jinan 250353, China

* Correspondence: authors: jlq@qlu.edu.cn (L.J.); xqh@qlu.edu.cn (Q.X.); Tel.: +86-531-89631168 (L.J.); Fax: +86-531-89631161 (L.J.)

Table S1. Compositions of the prepared hydrogels.

Hydrogels	Gel (g)	OCS (g)	TGA (mg)	Cur@Lipo (mL)	H_2O (mL)
GO	1.5	0.5			10
GOT-1	1.5	0.5	15		10
GOT-3	1.5	0.5	45		10
GOT-6	1.5	0.5	90		10
GOT-3/Cur@Lipo	1.5	0.5	45	5	5

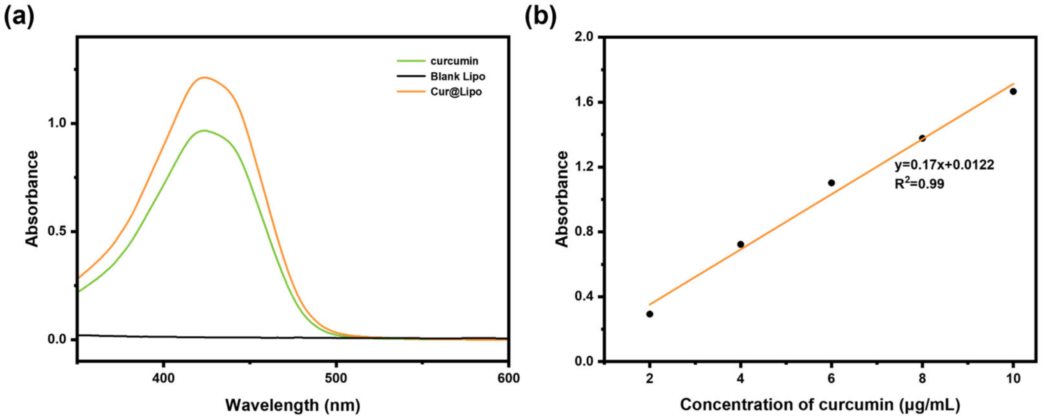

Figure S1. (**a**) UV-Vis absorption spectra of Cur, Blank Lipo, and Cur@Lipo; (**b**) Standard curve of curcumin at 425 nm.

Relative Crosslinking Extent of hydrogels

Relative crosslinking extent of GO and GOT hydrogels was determined using the ninhydrin assay. First, standard curve of glycine solutions was established. Subsequently, 2 mg of lyophilized hydrogel samples were weighed and dissolved in 1 mL of distilled water, followed by the addition of 2 mL of the ninhydrin reagent. The mixture was heated in a boiling water bath for 20 min. After cooling to room temperature, 5 mL of 50% (v/v) isopropanol was added for dilution. The absorbance of the resulting solution was measured at 570 nm using UV-2600 spectrophotometer (Shimadzu Corp., Japan). The relative crosslinking extent of hydrogels was calculated using the following formula [49]:

$$Relative\ crosslinking\ extent = \frac{C_0 - C_t}{C_0} \times 100\% \quad (S1)$$

where C_0 represents the free amino group content of the uncrosslinked sample, and C_t represents the free amino group content of the crosslinked sample.

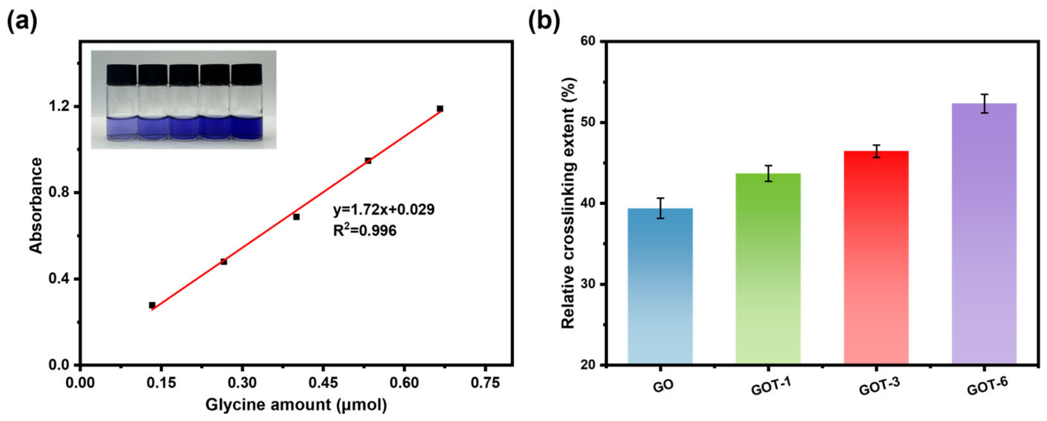

Figure S2. (**a**) Standard curve of glycine solutions; (**b**) Relative crosslinking extent of GO, GOT-1, GOT-3 and GOT-6 hydrogels.

Self-healing Efficiency of GOT-3/Cur@Lipo

The GOT-3/Cur@Lipo hydrogel with a diameter of 22 mm and a height of 5 mm was prepared, and one group was dyed using Rhodamine B solution for visual distinction. Each sample was evenly cut into two pieces, and the different colored hydrogels were brought into close contact at 37 °C for 1 h to observe their self-healing properties. To quantitatively evaluate the self-healing properties of GOT-3/Cur@Lipo hydrogel, frequency sweep measurements were performed on the original and healed hydrogels. Healing efficiency was determined using the following formula:

$$Healing\ efficiency = \frac{G'_{healed}}{G'_{original}} \times 100\% \quad (S2)$$

where $G'_{original}$ and G'_{healed} represent the storage modulus (G') of the hydrogel at an angular frequency of 1 rad/s before and after self-healing, respectively.

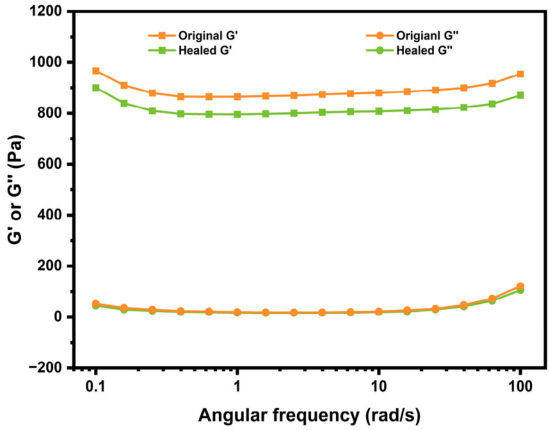

Figure S3. G′ and G″ of the GOT-3/Cur@Lipo hydrogel before and after self-healing.

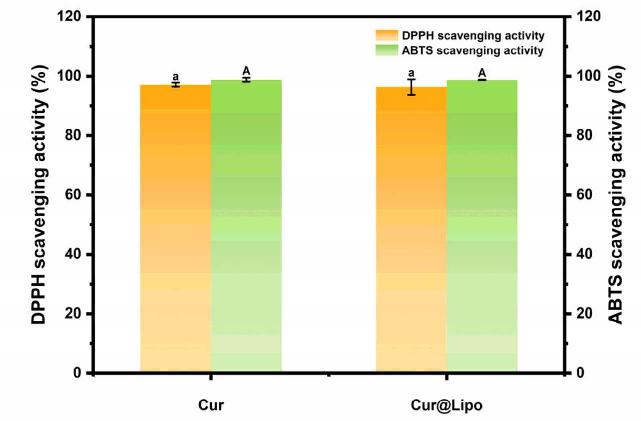

Figure S4. Antioxidant activities of free Cur and Cur@Lipo at equivalent curcumin concentrations. Different letters on the bars represent statistically significant differences ($p < 0.05$).

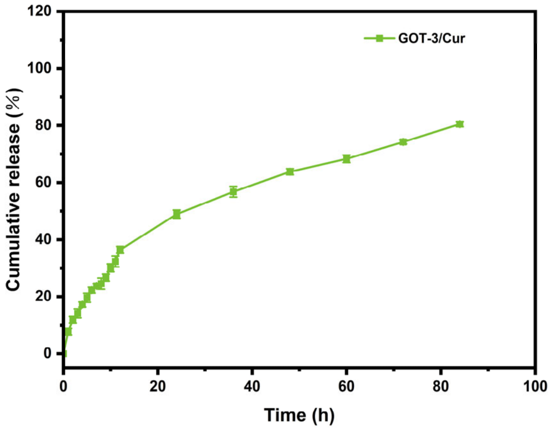

Figure S5. Cumulative release of curcumin from GOT-3/Cur hydrogels.

Movie S1. The injectability of GOT-3/Cur@Lipo hydrogel.

Movie S2. GOT-3/Cur@Lipo hydrogel continuously writes the letter “QLU” through a syringe.
